# First record of the introduced ladybird beetle, *Coccinella undecimpunctata* Linnaeus (1758), on South Georgia (sub‐Antarctic)

**DOI:** 10.1002/ece3.10513

**Published:** 2023-09-10

**Authors:** Pierre Tichit, Helen E. Roy, Peter Convey, Paul Brickle, Rosemary J. Newton, Wayne Dawson

**Affiliations:** ^1^ Department of Biosciences Durham University Durham UK; ^2^ UK Centre for Ecology & Hydrology Wallingford UK; ^3^ British Antarctic Survey (BAS), Natural Environment Research Council Cambridge UK; ^4^ Department of Zoology University of Johannesburg Auckland Park South Africa; ^5^ Biodiversity of Antarctic and Sub‐Antarctic Ecosystems (BASE) Santiago Chile; ^6^ Cape Horn International Center (CHIC) Puerto Williams Chile; ^7^ South Atlantic Environmental Research Institute (SAERI) Stanley Falkland Islands; ^8^ School of Biological Sciences (Zoology) University of Aberdeen Aberdeen UK; ^9^ Ecosystem Stewardship Royal Botanic Gardens Kew Ardingly UK

**Keywords:** biological invasion, biosecurity, Coccinellidae, Coleoptera, invasive alien species, non‐native species, sub‐Antarctic entomology, surveillance

## Abstract

Biological invasions represent a growing threat to islands and their biodiversity across the world. The isolated sub‐Antarctic island of South Georgia in the South Atlantic Ocean is a highly protected area that relies on effective biosecurity including prevention, surveillance and eradication to limit the risk of biological invasions. Based on an opportunistic field discovery, we provide the first report of an introduced ladybird beetle on South Georgia. All specimens discovered belong to the Eurasian species *Coccinella undecimpunctata* Linnaeus (1758) (Coleoptera: Coccinellidae). Tens of individuals of both sexes were discovered at a single location, indicating that the species may already be established on South Georgia. Transport connectivity with this site suggests that the species most likely arrived recently from the Falkland Islands as a stowaway on a ship. We discuss the implications of our discovery for the continued development of South Atlantic biosecurity.

## Graphical abstract

During a scientific expedition in South Georgia, we incidentally discovered the presence of the introduced ladybird beetle *Coccinella undecimpunctata*. The species may already be established and reproducing on the island and likely arrived from the Falkland Islands as a stowaway on a ship. This discovery has implications for the continued optimisation of South Atlantic biosecurity and global island conservation.

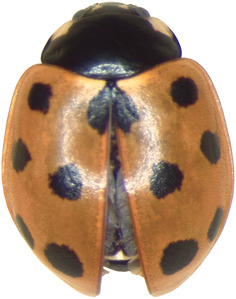

## 1 INTRODUCTION

Species introduced through human activities pose a major threat to islands and their biodiversity (Simberloff et al., 2013; Spatz et al., 2017). Unintentionally and intentionally introduced species can establish populations, spread across the landscape and become highly invasive with negative consequences for island communities, sometimes leading to extinctions of native species and radical transformations of the ecosystem (Gallardo et al., 2022; Pyšek et al., 2020; Seebens et al., 2021). There are numerous small and isolated oceanic islands located around the Antarctic continent (Selkirk, 2007). These sub‐Antarctic islands are highly vulnerable to biological invasions (Convey, 2007; Frenot et al., 2005), potentially because (1) native communities contain vacant niches as a result of geographical isolation and may thus have low resistance to invasion, (2) there has been an increase in human activities (e.g. tourism, scientific expeditions) likely associated with a higher propagule pressure of non‐native species, and (3) contemporary climate change may both destabilise native ecosystems and facilitate the establishment and spread of introduced species (Bergstrom & Chown, 1999; Convey & Lebouvier, 2009; Houghton et al., 2019; Hughes et al., 2019). Successful biosecurity measures are key to counteracting the growing challenge of biological invasions on sub‐Antarctic islands, by preventing introductions and managing species already present in a given area. Among biosecurity measures, prevention and early actions through rapid response are the most cost effective and have the greatest probability of success (Gallardo et al., 2022). Effective surveillance is a critical prerequisite to allow the early detection of new arrivals and the instigation of rapid responses (Berec et al., 2015; Latombe et al., 2017).

The Government of South Georgia and the South Sandwich Islands (GSGSSI) has adopted an ambitious environmental strategy to protect these islands, located in the South Atlantic sector of the Southern Ocean, south of the oceanic Antarctic polar front (GSGSSI, 2021). The entire island of South Georgia is a specially protected area (GSGSSI, 2022a) and any terrestrial activity is subject to some of the most rigorous biosecurity controls in the world (GSGSSI, 2022b). Invasive non‐native mammals (rats, mice, reindeer) have been eradicated from the Territory (GSGSSI, 2013; Martin & Richardson, 2019) and a strategy to eradicate or manage introduced vascular plants is in place (Black, 2022). Preventative measures include informing visitors and operators about the threat from biological invasions and the importance of biosecurity, and inspecting all incoming clothing, goods and equipment. In terms of surveillance, detection dogs, traps and baits are being used to detect any incursion of invasive rodents, while introduced arthropods are monitored by sticky traps placed across high‐risk areas and all habited buildings (Reid, 2019).

Among the approximately 6000 described species (Che et al., 2021) of ladybird beetles (Coccinellidae, hereafter simply referred as ladybird), several are invasive in many regions of the world (Brown et al., 2011). *Coccinella undecimpunctata* is established in North America (Smyth et al., 2013), Australia (Pope, 1988) and New Zealand (Galbreath & Cameron, 2015). It has not been recorded in the natural environment on South Georgia or elsewhere in the sub‐Antarctic (Leihy et al., 2023), although Houghton et al. (2016) note that it has been found in association with cargo on Antarctic logistics vessels. Here we provide the first report of the presence of the Eurasian ladybird, *C. undecimpunctata* Linnaeus (1758) (Coleoptera: Coccinellidae) on South Georgia.

## 2 MATERIALS AND METHODS

### 2.1 Sample collection

**FIGURE 1:**
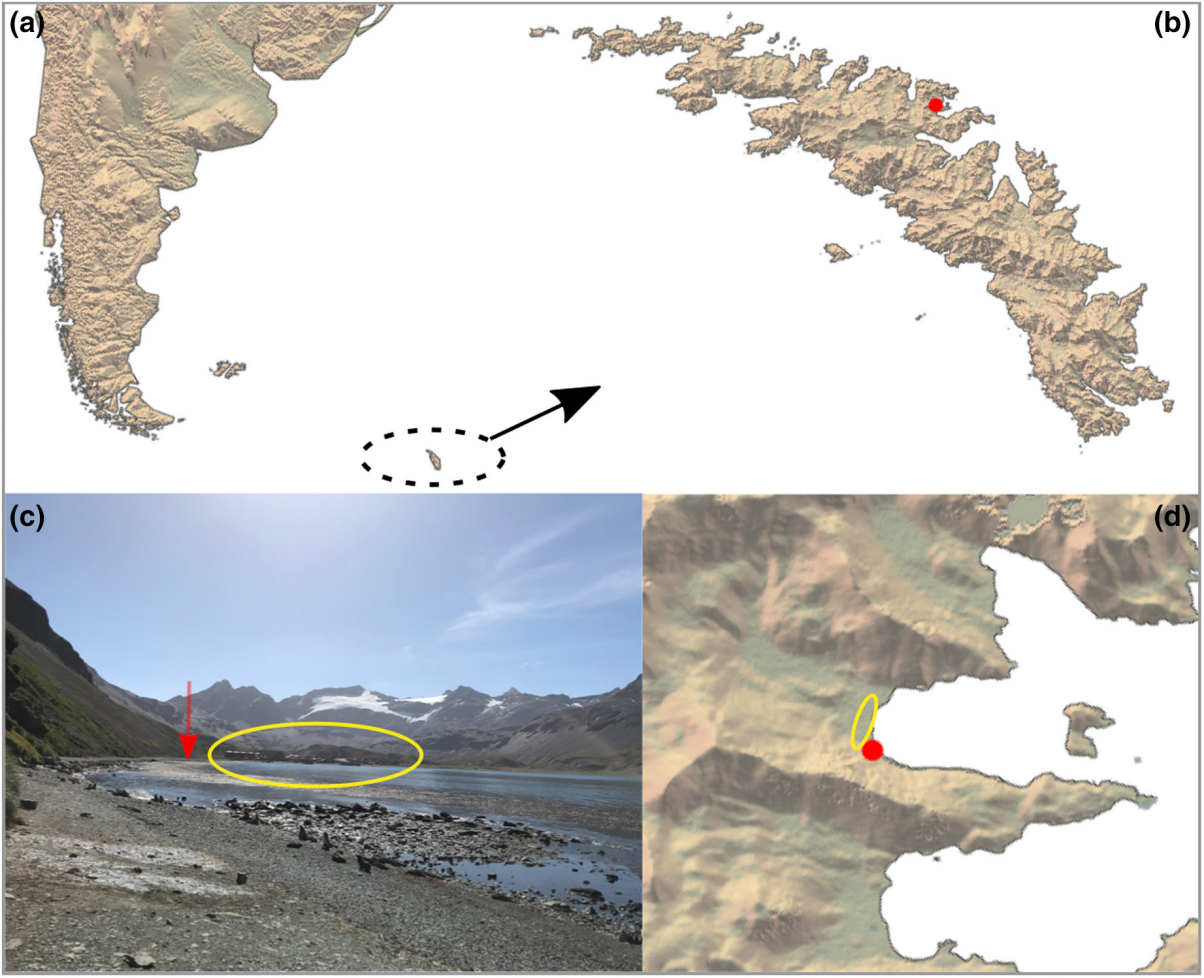
(a, b) Location of the record in the north‐central part of South Georgia (red point). (c, d) Location of the record in Stromness Bay (red point and arrow). The abandoned whaling station is highlighted in yellow.

Live ladybirds were discovered opportunistically and collected while carrying out biological survey work at the southern side of Stromness Bay (South Georgia, Figure 1). The location was approximately 300 m from the derelict Stromness whaling station (Figure 1c,d). Sample collections were made by two researchers at around 1200 (local time) on 24 and 30 January 2023. At the time of collection there was a light breeze with an air temperature of 8–10°C, to be placed in the context of a mean standard air temperature of 2.6°C (min = −22°C, max = 26°C) recorded between 1991 and 2021 at King Edward Point Meteorological Station. Hand searches were made among stones and crevices within a radius of a few metres from the initial point of discovery. The total search duration was approximately 12 min. Specimens were collected gently by hand or with soft forceps and immediately transferred to 96% ethanol for preservation.

### 2.2 Sample processing and identification

Morphological features allowing the identification of preserved specimens were observed under a stereomicroscope. To obtain good quality images of the external morphology, three individuals were dried at ambient temperature. An elytron, wing, mandible, antenna, as well as a male and a female reproductive system were dissected using fine forceps and micro scissors and photographed with an integrated camera IC90 E (Leica Microsystems). Cutting through the posterior tip of the abdomen to expose the reproductive system enabled determination of the sex of most individuals. For a few specimens that were kept intact (*n* = 6), sex determination relied on the sexual dimorphism of the 8th abdominal segment: males have a “notch” on the posterior margin of the sternite (Stellwag & Losey, 2014). The width of the pronotum and head were measured on calibrated pictures of specimens using the software ImageJ (Schneider et al., 2012).

### 2.3 Data analysis and visualisation

All statistical analyses were performed in R (R Core Team, 2022). Two‐sided Welch *t*‐tests were used to investigate if the widths of the pronotum and of the head differed between sexes. Maps of South Georgia were based on topographic data from thematicmapping.org and South Georgia GIS (2023). Figures were assembled in Inkscape (Inkscape Project, 2020). Unless otherwise specified, ‘*n*’ represents the number of individual beetles and values are given as mean ± 95% confidence interval.

## 3 RESULTS

In January 2023, we recorded the presence of ladybirds on the southern side of Stromness Bay, South Georgia (Figure 1). Despite the limited search effort (two researchers for about 12 min) we observed at least 23 live individuals of which 18 were collected. The detectable presence of ladybirds appeared restricted to an area of a few square meters (−54°9′42.451″, −36°42′38.588″). While we did not perform systematic searches around the sampling area, we did not detect any other specimens during surveys in 2022 (9 days) or 2023 (28 days) of terrestrial arthropods using pitfall traps and hand searches in Stromness, Husvik, Leith, Fortuna and King Haakon Bay nor across the Thatcher and Barff Peninsulas. The ladybirds were found at the base of a north‐facing scree slope approximately 10 m from the shore. The habitat consisted of rocks with very sparse vegetation dominated by annual meadow grass (*Poa annua*, itself a non‐native species). Antarctic fur seals (*Arctocephalus gazella*) move between the sea and their resting areas via the sampling location and a group of moulting king penguins (*Aptenodytes patagonicus*) was present in the vicinity. Consequently, organic debris and feathers were abundant below and between rocks in the sampled area.

**FIGURE 2:**
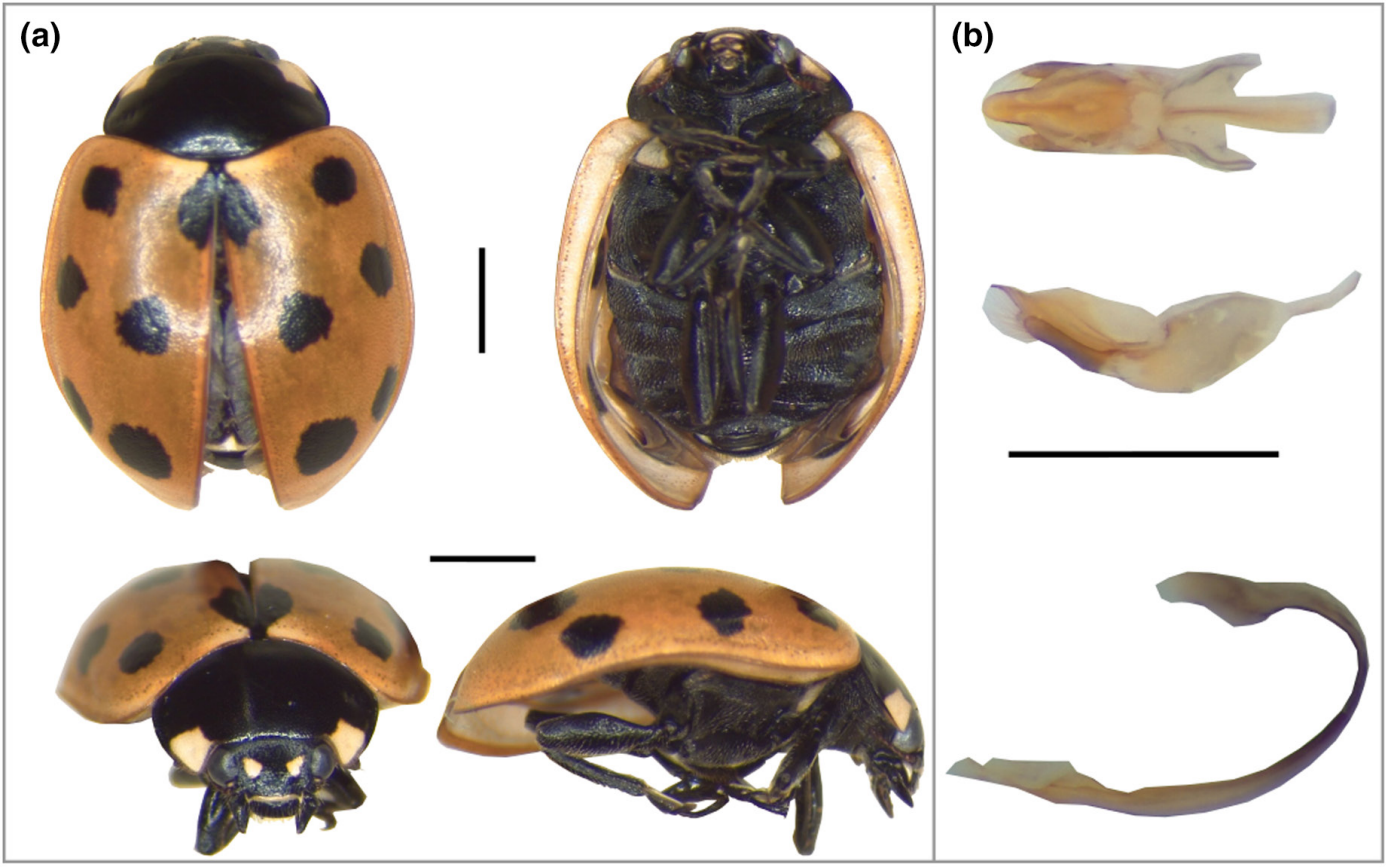
(a) External morphology of *Coccinella undecimpunctata* (male) collected on South Georgia. Scale bar = 1 mm. (b) Details of male reproductive system: tegmen (top) and sipho (bottom). Scale bar = 1 mm.

All specimens were identified as the 11‐spot ladybird *C. undecimpunctata* Linnaeus (1758). Specimens had small and elongate bodies (length ≈5 mm, Figure 2), 11 dark round spots on the red‐orange elytra, two distinct small white spots between the eyes and one white spot on each anterior margin of the pronotum, as described by Schaeffer (1912). The shape of the male reproductive system, in particular the sipho (Figure 2b), corresponded with previous descriptions of *C. undecimpunctata* (Badrawy et al., 2009; Hawkes & Marriner, 1927). The species identity was established independently based on Figure 2 by two of the authors (HER and PT).

**FIGURE A1:**
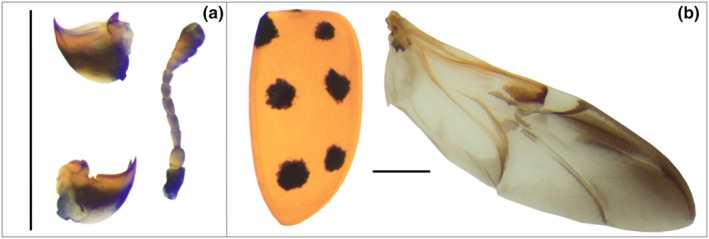
(a) Right mandible and antenna. (b) Right elytron and wing. Scale bar = 1 mm.

**FIGURE A2:**
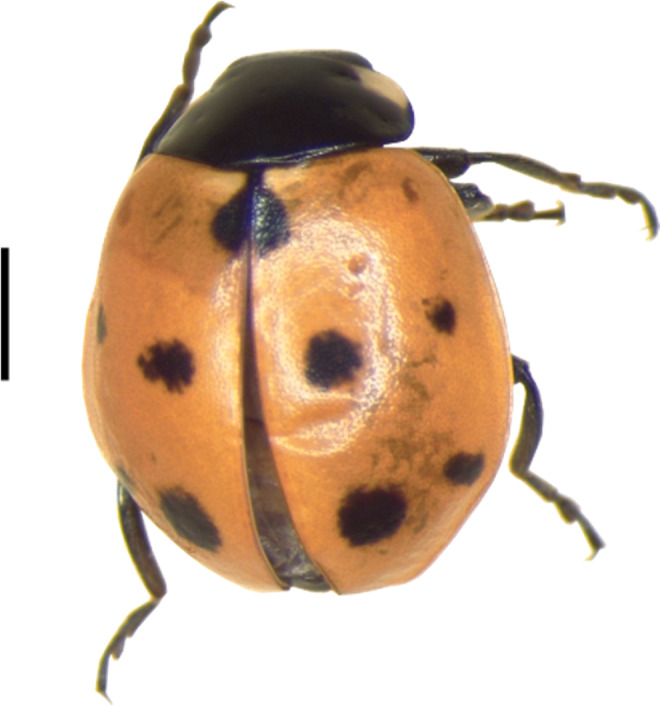
Example of a specimen with reduced humeral spot. Scale bar = 1 mm.

The sex ratio of the collected specimens was close to 50:50, with eight females and 10 males. There was little variation in two proxies of body size – the width of the pronotum (2.0 ± 0.1 mm) and of the head (1.1 ± 0.05 mm) – and these were not significantly related to sex (two‐sided Welch *t*‐test; pronotum: *t* = 0.36, df = 15, *p* = .72; head: *t* = −0.49, df = 16, *p* = .63; Table A1). Elytral patterns varied across specimens, with the humeral spot sometimes being very reduced (*n* = 3, Figures A1 and A2).

## 4 DISCUSSION

We provide the first record of the ladybird *C. undecimpunctata* Linnaeus (1758) on the sub‐Antarctic island of South Georgia. There is little doubt that the presence of this Eurasian species is due to human activity. *C. undecimpunctata* may be reproducing on South Georgia given the presence of both sexes and the number of individuals found relative to search effort. However, the species was detected only in a small area and not at any other sampling site on the northern peninsulas nor in King Haakon Bay and may therefore be at an early stage of biological invasion (Gallardo et al., 2022).

The ladybirds introduced to South Georgia most likely originated from introduced populations in the Falkland Islands, the closest land mass where the species has been reported (Robinson, 1984) and from which most shipping traffic to the island originates. To reach South Georgia from the Falkland Islands, the beetles would have had to cross more than 1000 km of ocean, a distance that ladybirds have not been reported to disperse via natural means. Moreover, ladybirds have been intercepted in high numbers on ships (Minchin, 2010), *C. undecimpunctata* has repeatedly been reported among cargo in the Antarctic region (Bergstrom et al., 2014; Houghton et al., 2016) and can undergo dormancy to survive adverse environmental conditions (Brown et al., 2011), so we suggest that the most likely introduction pathway is maritime transport.

The introduced ladybird population is located in Stromness Bay where the most prominent human activity – and hence the most likely introduction pathway – is tourism. Approximately 4000 passengers and support personnel land in Stromness Bay every year (GSGSSI, 2019), although the bay is also visited by scientists based at King Edward Point research station and visiting research vessels, and by GSGSSI staff responsible for inspection of the abandoned whaling station. The small beetles may have “hitch‐hiked” on a cruise ship and dispersed to the shore of South Georgia on their own or as “stowaway” in personal clothing during landings, escaping the strict biosecurity procedures imposed on cruise ships by GSGSSI.

In other regions, introduced ladybirds negatively impact native communities (Evans et al., 2011), which underlines the necessity of developing a management strategy for *C. undecimpunctata* on South Georgia. As a prerequisite to inform management, we recommend assessing the state and extent of the located population during the austral spring of 2023/24. Ladybirds are highly mobile insects capable of walking, flying and being wind‐blown (Brown et al., 2011). It is key to recognise this high mobility by carrying out systematic searches in the wider landscape, starting with favourable habitat in lowland coastal areas (Benham & Muggleton, 1976), to establish the species' distribution. If the population is restricted to one or a few small patches, complete eradication may be possible. While eradicating highly mobile insects is challenging, successes on other islands show that it is feasible (Simberloff et al., 2018). A detailed cost–benefit and environmental impact assessment would be required prior to any management attempt on South Georgia. Despite the proven effectiveness of traps or insecticides to eradicate small ladybird populations (Kenis et al., 2008), non‐target effects on native ecosystems must be considered.

South Georgia is a potential stepping‐stone for future introductions to the Scotia Arc archipelagos and Antarctic Peninsula, via maritime traffic. To avoid further human‐aided dispersal of ladybirds within and from South Georgia, it is crucial that rigorous biosecurity measures are maintained. Improved awareness about the potential for introduced arthropods among tourism and other operators visiting South Georgia is vital to maximise chances of detecting arthropod stowaways during inspections of clothing and cargo. Ladybirds are brightly‐coloured and easily recognised but other stowaways may be more cryptic. Light traps, sticky traps and vane traps (Kenis et al., 2008) placed on the inside and outside of incoming ships would increase the detection capacity of invertebrates. Finally, inter‐regional cooperation between tourism operators, GSGSSI and other regional stakeholders to publish past and future interception records would be valuable to strengthen the early response capacity.

## AUTHOR CONTRIBUTIONS

**Pierre Tichit:** Conceptualization (lead); data curation (lead); formal analysis (lead); investigation (lead); methodology (lead); project administration (supporting); software (lead); supervision (supporting); validation (lead); visualization (lead); writing – original draft (lead); writing – review and editing (lead). **Helen E. Roy:** Formal analysis (supporting); validation (supporting); writing – review and editing (supporting). **Peter Convey:** Conceptualization (supporting); funding acquisition (supporting); investigation (supporting); methodology (supporting); project administration (supporting); writing – review and editing (supporting). **Paul Brickle:** Conceptualization (supporting); funding acquisition (supporting); methodology (supporting); project administration (supporting); resources (supporting); writing – review and editing (supporting). **Rosemary J. Newton:** Conceptualization (supporting); funding acquisition (supporting); methodology (supporting); project administration (supporting); writing – review and editing (supporting). **Wayne Dawson:** Conceptualization (supporting); data curation (supporting); formal analysis (supporting); funding acquisition (lead); methodology (supporting); project administration (lead); resources (lead); supervision (lead); validation (supporting); writing – original draft (supporting); writing – review and editing (supporting).

## CONFLICT OF INTEREST STATEMENT

The authors declare no competing interests.

## Data Availability

Pictures of specimens under the stereomicroscope and associated information for size calibration are available on Dryad (https://doi.org/10.5061/dryad.1zcrjdfzh).

## APPENDIX 1

**TABLE A1:** Sex, pronotum width and head width of all collected specimens (*n* = 18).

Individual	Sex	Pronotum width (mm)	Head width (mm)
1	Female	1.95	1.07
2	Male	2.06	1.22
3	Male	1.79	1.07
4	Male	1.89	1.12
5	Female	1.97	1.10
6	Male	2.11	1.17
7	Male	2.00	1.08
8	Female	1.80	1.08
9	Male	2.05	1.18
10	Female	2.06	1.20
11	Male	1.86	1.09
12	Female	2.17	1.15
13	Male	2.01	1.17
14	Male	2.16	1.21
15	Female	2.04	1.15
16	Male	2.00	1.17
17	Female	1.99	1.15
18	Female	2.12	1.18
